# A Retrospective Study of Outcomes of Surgical Management of Severe Laryngomalacia

**DOI:** 10.7759/cureus.105540

**Published:** 2026-03-20

**Authors:** Satish Kumar K, Prem Kumar P, Bincy Joseph, Sahana Raju, Afshan Fathima

**Affiliations:** 1 Otorhinolaryngology - Head and Neck Surgery, PES University Institute of Medical Sciences and Research, Bengaluru, IND; 2 Pediatric Otorhinolaryngology, Indira Gandhi Institute of Child Health, Bengaluru, IND; 3 Otorhinolaryngology, Kerala Health Services, Kannur, IND; 4 Otorhinolaryngology, Bangalore Medical College and Research Institute, Bengaluru, IND

**Keywords:** aryepiglottoplasty, epiglottopexy, laryngomalacia, laryngotracheobronchoscopy, neonatal stridor

## Abstract

Background: Laryngomalacia is the most common cause of congenital stridor in infants. While most cases are mild and self-limiting, some develop severe disease requiring surgical intervention due to airway obstruction, feeding difficulties, or failure to thrive. This study evaluates the outcomes of surgical management for severe laryngomalacia at a tertiary care pediatric center.

Methods: A retrospective review was conducted of 56 children with severe laryngomalacia who underwent surgery between January 2013 and January 2023. Patients with secondary airway lesions or significant comorbidities were excluded. Laryngotracheobronchoscopy findings classified patients into type 1-3 or combined types. Surgical interventions included aryepiglottoplasty, epiglottopexy, and resection of the arytenoid mucosa, performed using conventional cold steel techniques. Postoperative outcomes assessed included relief from stridor, extubation timing, need for second intervention or tracheostomy, and complications. Statistical analysis identified factors associated with outcomes.

Results: The mean age was 3.67 ± 5.03 months; 58.9% were males. Type 2 laryngomalacia was most common (41.1%). Aryepiglottoplasty was the primary procedure in 41.8% of cases. Postoperatively, 80.4% achieved relief from stridor, 67.9% were extubated within 24 hours, and 14.3% required a second intervention. Tracheostomy was performed in 5.4% of patients, all with type 3 laryngomalacia. Younger age and absence of failure to thrive were significantly associated with favorable outcomes (p < 0.05). Delayed extubation and second interventions were more frequent in older infants and those with failure to thrive. No intraoperative or postoperative complications were observed.

Conclusion: Surgical management of severe laryngomalacia is safe and effective, with high success rates and minimal morbidity. Early intervention and absence of failure to thrive predict better outcomes, while type 3 laryngomalacia is a strong predictor for tracheostomy. Individualized anatomical assessment remains crucial for optimal surgical planning.

## Introduction

A child born with stridor is a harrowing experience for the parents, stealing the joy of welcoming a newborn into the family. Laryngomalacia is the most common congenital abnormality of the larynx, accounting for about 60% to 75% of congenital stridor cases [1]. Although its pathogenesis is not fully understood in this condition, there is a collapse of supraglottic tissues during inspiration, generating a high-frequency inspiratory stridor that is exacerbated in the supine position during feeding, agitation, and crying. This stridor usually appears in the first two weeks of life, with an incidence peak at around six months and spontaneous resolution in 90% of cases by the second year of life [1].

The severity of laryngomalacia can be mild, moderate, or severe. Most cases are mild and present with inspiratory stridor with a coordinated suck-swallow-breathe sequence; such cases do not require therapeutic intervention. Mild laryngomalacia resolves spontaneously within one to two years in 70% of patients. Moderate to severe cases may be complicated by feeding difficulties, gastroesophageal reflux, failure to thrive, cyanosis, intermittent complete obstruction, or cardiac failure [2-5]. A total of 10-15% of laryngomalacia cases require intervention to relieve respiratory obstruction [6].

Surgical management of laryngomalacia is indicated only in severe cases, and supraglottoplasty is the surgical treatment of choice [2]. Supraglottoplasty may be performed using micro-laryngeal instruments (cold knife technique) or CO2 laser or coblation [7]. The present study aims to review the outcomes of surgical management in severe laryngomalacia at our tertiary care center and to assess its effectiveness in relieving airway obstruction and improving clinical outcomes.

## Materials and methods

This retrospective study covers a period of 10 years, from January 2013 to January 2023, at a single center. After obtaining the institutional review board's clearance, those fulfilling the inclusion criteria were enrolled in the study. A total of 56 cases were studied.

Medical records were accessed, and details of history and clinical examination findings were obtained. Children presenting with persistent stridor, feeding difficulty, failure to thrive, and/or cyanosis were considered for the study. Laryngotracheobronchoscopy findings of these patients were noted. Based on the history and laryngotracheobronchoscopy findings, patients were divided into mild, moderate, and severe cases of laryngomalacia. Only severe cases of laryngomalacia were included in the study.

Patients with secondary airway lesions like vocal cord palsy, laryngeal web, subglottic stenosis, and severe trachea-bronchomalacia were excluded. Patients with comorbidities like progressive neurological disorders, cardiac diseases, and bleeding diathesis were also excluded. Cases with incomplete data were also excluded from the study.

The type of laryngomalacia in individual patients was further noted. Details of the surgical procedure performed, i.e., aryepiglottoplasty, epiglottopexy, and resection of arytenoid mucosa, were obtained from operative notes. It was noted that all procedures were done by the conventional cold steel method. Postoperative outcome was assessed.

The type of laryngomalacia was categorized based on endoscopy findings as follows: type 1: inward collapse of the aryepiglottic folds on inspiration; type 2: curled tubular epiglottis with shortened aryepiglottic folds, which collapse circumferentially on inspiration; type 3: an overhanging epiglottis that collapses posteriorly, obstructing the laryngeal inlet on inspiration.

Surgical procedure

All procedures were done under general anesthesia with a nasopharyngeal airway. The larynx was exposed with an intubating laryngoscope and visualized using a rigid endoscope during spontaneous respiration. Surgical procedures were carried out using conventional cold steel instruments and electrocautery.

The following three surgical procedures were used: (1) aryepiglottoplasty: shortened aryepiglottic folds were released by making a cut with microlaryngeal scissors closer to the epiglottis; (2) epiglottopexy: adhesion between the lingual surface of the epiglottis and base of the tongue is achieved by creating a raw surface with electrocautery; (3) resection of arytenoid mucosa: redundant arytenoid mucosa is held with forceps, and adequate resection is done with microlaryngeal scissors.

Type 1 laryngomalacia cases underwent resection of the arytenoid mucosa. Type 2 laryngomalacia cases underwent aryepiglottoplasty. Type 3 laryngomalacia cases underwent epiglottopexy. Cases with combined types underwent a combination of these three surgical procedures.

The outcome of surgery was evaluated based on (1) extubation within 24 hours, (2) delayed extubation, (3) relief from stridor, (4) requirement of a second intervention, (5) tracheostomy performed, and (6) postoperative complications.

Data were entered and analyzed using Microsoft Excel (Microsoft Corporation, Redmond, WA).

## Results

**Figure 1:**
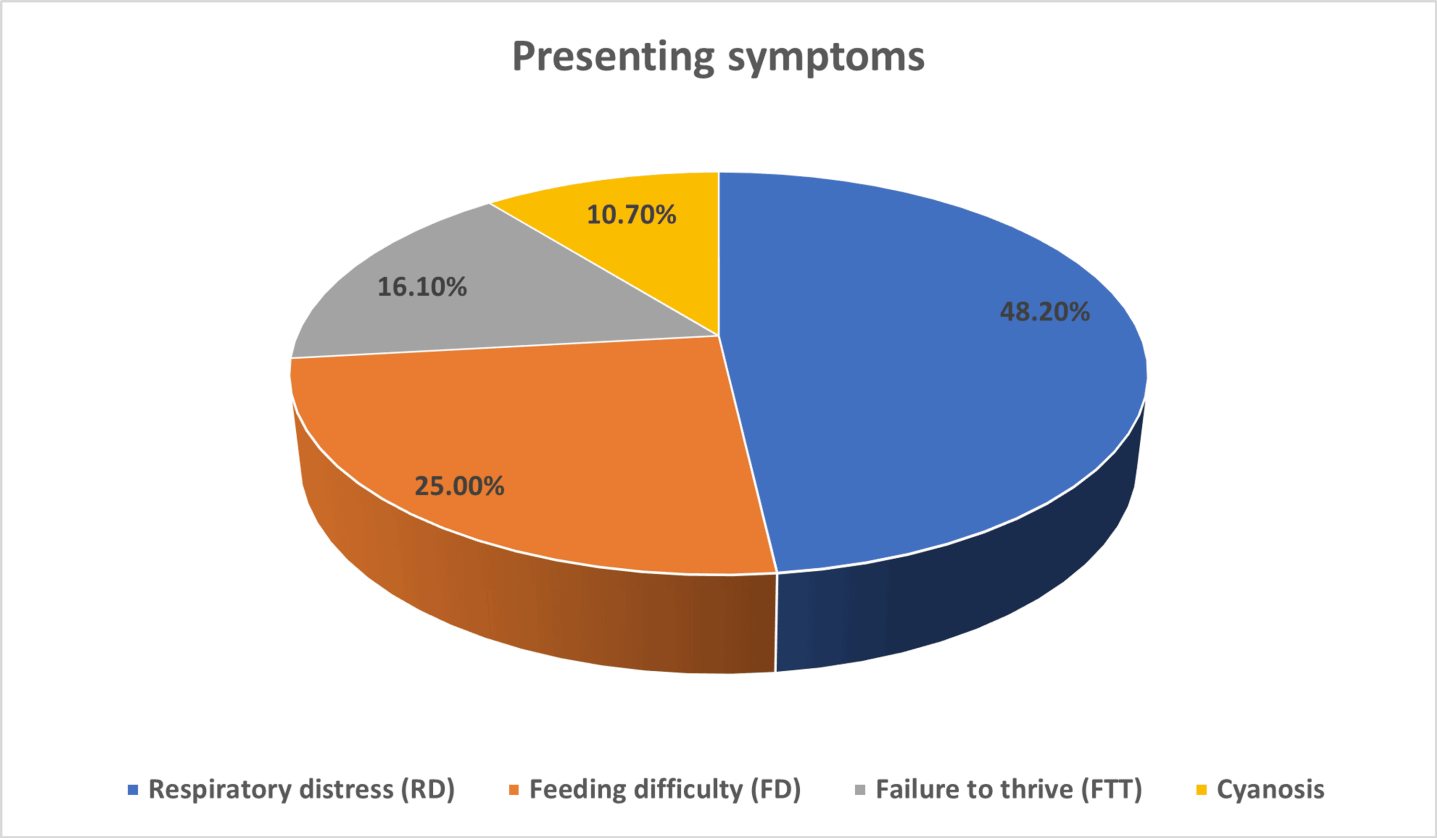
Presenting symptoms.

The mean age of the study subjects was 3.67 (± 5.03) months, while the median age was 2.0 months. The majority of the subjects belonged to the infant age categories, with 41.1% being between zero and one month old and 50.0% between one and 10 months old. In terms of gender distribution, there were more males (58.9%) than females (41.1%) (Table 1). The most common presenting symptom was respiratory distress, affecting 48.2% of the children, followed by feeding difficulty in 25.0%, failure to thrive in 16.1%, and cyanosis in 10.7%, as depicted in Figure 1.

**Table 1:** Baseline demographic and clinical characteristics of study participants (n = 56).

Variable	Category	n (%)/value
Age (months)	Mean ± standard deviation	3.67 ± 5.03
	Median	2.0
	Range	0.2-25.0
Age categories	0-1 month	23 (41.1%)
	1-10 months	28 (50.0%)
	10-20 months	4 (7.1%)
	>20 months	1 (1.8%)
Sex	Male	33 (58.9%)
	Female	23 (41.1%)
Presenting symptom	Respiratory distress	27 (48.2%)
	Feeding difficulty	14 (25.0%)
	Failure to thrive	9 (16.1%)
	Cyanosis	6 (10.7%)

**Figure 2:**
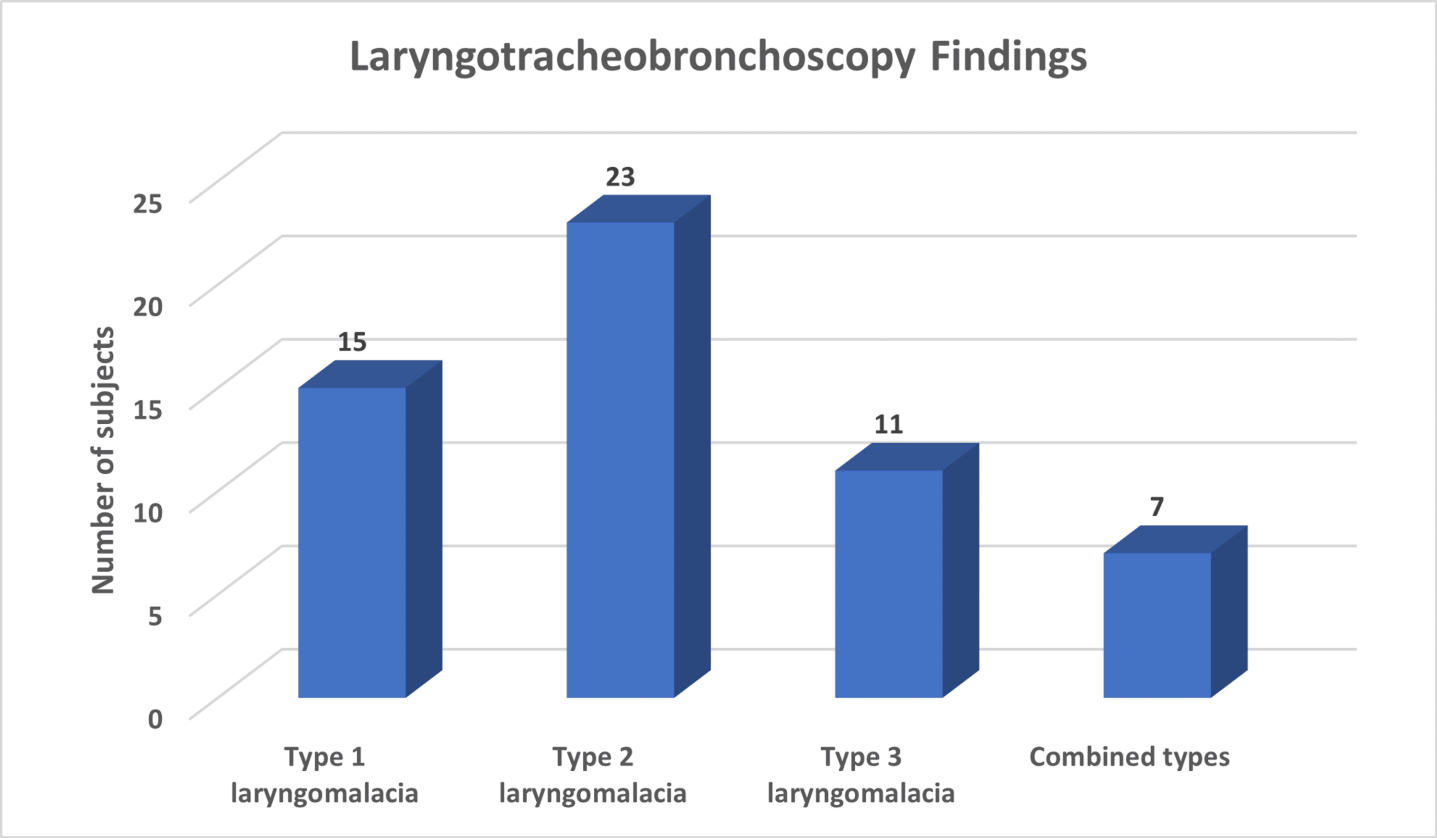
Laryngotracheobronchoscopy findings.

The most frequent anatomical finding was type 2 laryngomalacia, observed in 41.1% of cases, followed by type 1 in 26.8% and type 3 in 19.6%. Combined types were seen in 12.5% of patients, as depicted in Table 2 and Figure 2. Regarding surgical management, aryepiglottoplasty was the primary procedure for 41.8% of the study subjects. Resection of the arytenoid mucosa was performed in 27.3% of cases, and epiglottopexy in 14.5%, with combined surgical approaches utilized for the remaining patients.

**Table 2:** Laryngotracheobronchoscopic findings and surgical procedures (n = 56).

Variable	Category	n (%)
Laryngotracheobronchoscopy findings	Type 1 laryngomalacia	15 (26.8%)
	Type 2 laryngomalacia	23 (41.1%)
	Type 3 laryngomalacia	11 (19.6%)
	Combined types	7 (12.5%)
Surgery performed	Aryepiglottoplasty	23 (41.8%)
	Epiglottopexy	8 (14.5%)
	Resection of the arytenoid mucosa	15 (27.3%)
	Aryepiglottoplasty with epiglottoplasty	6 (10.9%)
	Aryepiglottoplasty with resection of the arytenoid mucosa	2 (3.6%)
	Aryepiglottoplasty with epiglottoplasty with resection of the arytenoid mucosa	1 (1.8%)

**Figure 3:**
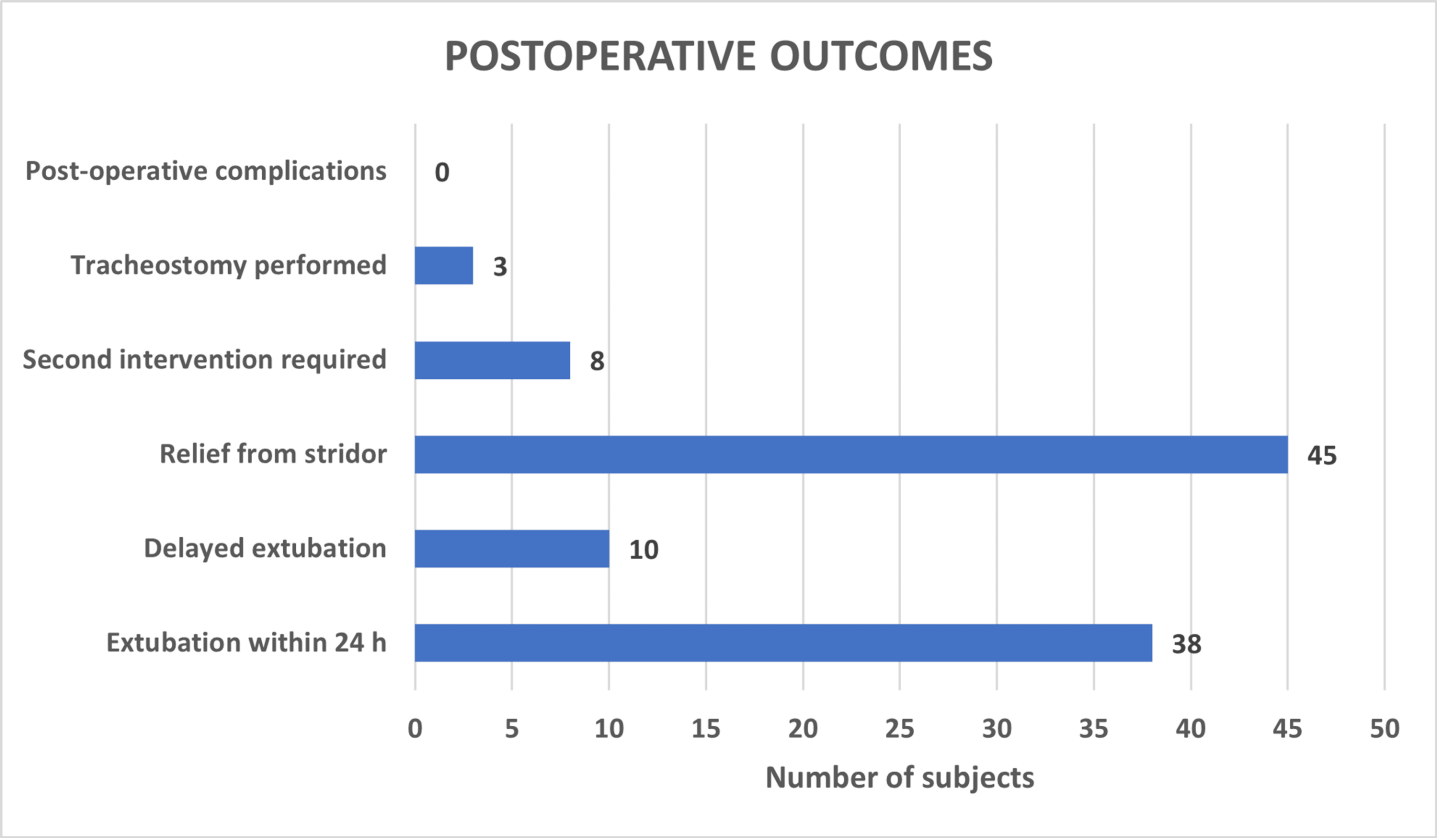
Postoperative outcomes.

Positive results were observed in the majority of cases, with 80.4% of patients achieving relief from stridor and 67.9% undergoing successful extubation within 24 hours. Conversely, delayed extubation occurred in 17.9% of cases. The proportion of re-intervention was 14.3%, and only three patients (5.4%) required a tracheostomy. The study recorded nil postoperative complications. Postoperative outcomes data have been depicted in Table 3 and Figure 3.

**Table 3:** Postoperative outcomes (n = 56).

Outcome	Yes, n (%)	No, n (%)
Extubation within 24 hours	38 (67.9%)	18 (32.1%)
Delayed extubation	10 (17.9%)	46 (82.1%)
Relief from stridor	45 (80.4%)	11 (19.6%)
Second intervention required	8 (14.3%)	48 (85.7%)
Tracheostomy performed	3 (5.4%)	53 (94.6%)
Postoperative complications	0 (0%)	56 (100%)

Patient age was a significant factor (p = 0.003), with patients who achieved relief from stridor being younger (mean = 2.56 months) compared to those who did not (mean = 8.23 months). Clinical presentation also showed significance (p = 0.031). Notably, failure to thrive was present in 45.5% of the group that failed to find relief from stridor. Additionally, anatomical findings were significantly associated with outcomes (p = 0.034), whereas sex and the specific type of surgery performed did not show a statistically significant correlation with stridor relief (Table 4).

**Table 4:** Factors associated with relief from stridor (n = 56).

Variable	Category	Relief present, n (%) (n = 45)	Relief absent, n (%) (n = 11)	Test	p-value
Age (months)	Mean ± standard deviation	2.56 ± 3.30	8.23 ± 7.92	Mann-Whitney	0.003
Sex	Male	25 (55.6)	8 (72.7)	Chi-square	0.299
	Female	20 (44.4)	3 (27.3)		
Presenting symptom	Respiratory distress	24 (53.3)	3 (27.3)	Chi-square	0.031
	Feeding difficulty	12 (26.7)	2 (18.2)		
	Failure to thrive	4 (8.9)	5 (45.5)		
	Cyanosis	5 (11.1)	1 (9.1)		
Laryngotracheobronchoscopy findings	Type 1 laryngomalacia	11 (24.4)	4 (36.4)	Chi-square	0.034
	Type 2 laryngomalacia	22 (48.9)	1 (9.1)		
	Type 3 laryngomalacia	6 (13.3)	5 (45.5)		
	Combined	6 (13.3)	1 (9.1)		
Surgery performed	Aryepiglottoplasty	21 (46.7)	2 (18.2)	Chi-square	0.385
	Epiglottopexy	6 (13.3)	2 (18.2)		
	Resection of the arytenoid mucosa	11 (24.4)	4 (36.4)		
	Combined	7 (15.6)	3 (27.3)		

The data indicate that older age is significantly associated with delayed extubation (p = 0.044), with the delayed group having a mean age of 5.84 months compared to 3.20 months for the non-delayed group. Other variables, including sex, presenting symptoms, anatomical laryngotracheobronchoscopy findings, and surgery type, did not show statistical significance regarding the likelihood of delayed extubation (Table 5).

**Table 5:** Factors associated with delayed extubation (n = 56).

Variable	Category	Delayed present, n (%) (n = 10)	Delayed absent, n (%) (n = 46)	Test	p-value
Age (months)	Mean ± standard deviation	5.84 ± 6.02	3.20 ± 4.73	Mann-Whitney	0.044
Sex	Male	6 (60.0)	27 (58.7)	Chi-square	0.939
	Female	4 (40.0)	19 (41.3)		
Presenting symptom	Respiratory distress	3 (30.0)	24 (52.2)	Chi-square	0.333
	Feeding difficulty	2 (20.0)	12 (26.1)		
	Failure to thrive	3 (30.0)	6 (13.0)		
	Cyanosis	2 (20.0)	4 (8.7)		
Laryngotracheobronchoscopy findings	Type 1 laryngomalacia	3 (30.0)	12 (26.1)	Chi-square	0.462
	Type 2 laryngomalacia	2 (20.0)	21 (45.7)		
	Type 3 laryngomalacia	3 (30.0)	8 (17.4)		
	Combined	2 (20.0)	5 (10.9)		
Surgery performed	Aryepiglottoplasty	2 (20.0)	21 (45.7)	Chi-square	0.321
	Epiglottopexy	3 (30.0)	5 (10.9)		
	Resection of the arytenoid mucosa	3 (30.0)	12 (26.1)		
	Combined	2 (20.0)	8 (17.4)		

There was a highly significant association between laryngotracheobronchoscopic findings and the need for tracheostomy (p = 0.005). Specifically, all three patients (100%) who required a tracheostomy were diagnosed with type 3 laryngomalacia. Other factors, including age, sex, presenting symptoms, and the type of surgery performed, were not statistically significant predictors for needing a tracheostomy (Table 6).

**Table 6:** Factors associated with tracheostomy (n = 56).

Variable	Category	Tracheostomy present, n (%) (n = 3)	Tracheostomy absent, n (%) (n = 53)	Test	p-value
Age (months)	Mean ± standard deviation	12.2 ± 12.3	3.19 ± 4.07	Mann-Whitney	0.341
Sex	Male	2 (66.7)	31 (58.5)	Fisher	0.779
	Female	1 (33.3)	22 (41.5)		
Presenting symptom	Respiratory distress	1 (33.3)	26 (49.1)	Fisher	0.097
	Feeding difficulty	0 (0)	14 (26.4)		
	Failure to thrive	2 (66.7)	7 (13.2)		
	Cyanosis	0 (0)	6 (11.3)		
Laryngotracheobronchoscopy findings	Type 1 laryngomalacia	0 (0)	15 (28.3)	Fisher	0.005
	Type 2 laryngomalacia	0 (0)	23 (43.4)		
	Type 3 laryngomalacia	3 (100)	8 (15.1)		
	Combined	0 (0)	7 (13.2)		
Surgery performed	Aryepiglottoplasty	0 (0)	23 (43.4)	Fisher	0.066
	Epiglottopexy	1 (33.3)	7 (13.2)		
	Resection of the arytenoid mucosa	0 (0)	15 (28.3)		
	Combined	2 (66.7)	8 (15.1)		

Age showed a statistically significant association (p = 0.001), with patients requiring further intervention being considerably older (mean = 9.38 months) compared to those who did not (mean = 2.72 months). The initial presenting symptom was also statistically significant (p = 0.039). Notably, 50% of the patients who required a second intervention had initially presented with failure to thrive. Sex, anatomical findings, and the surgery type were not statistically significant factors (Table 7).

**Table 7:** Factors associated with second intervention (n = 56).

Variable	Category	Intervention present, n (%) (n = 8)	No intervention, n (%) (n = 48)	Test	p-value
Age (months)	Mean ± standard deviation	9.38 ± 8.58	2.72 ± 3.49	Mann-Whitney	0.001
Sex	Male	7 (87.5)	26 (54.2)	Chi-square	0.076
	Female	1 (12.5)	22 (45.8)		
Presenting symptom	Respiratory distress	2 (25.0)	25 (52.1)	Chi-square	0.039
	Feeding difficulty	1 (12.5)	13 (27.1)		
	Failure to thrive	4 (50.0)	5 (10.4)		
	Cyanosis	1 (12.5)	5 (10.4)		
Laryngotracheobronchoscopy findings	Type 1 laryngomalacia	3 (37.5)	12 (25.0)	Chi-square	0.287
	Type 2 laryngomalacia	1 (12.5)	22 (45.8)		
	Type 3 laryngomalacia	3 (37.5)	8 (16.7)		
	Combined	1 (12.5)	6 (12.5)		
Surgery performed	Aryepiglottoplasty	2 (25.0)	21 (43.8)	Chi-square	0.735
	Epiglottopexy	1 (12.5)	7 (14.6)		
	Resection of the arytenoid mucosa	3 (37.5)	12 (25.0)		
	Combined	2 (25.0)	8 (16.7)		

This outcome was significantly associated with younger patient age (p = 0.002), with the early extubation group having a mean age of 2.41 months compared to 6.33 months for those with delayed extubation. Other clinical variables, such as sex, presenting symptoms, anatomical findings, and surgical technique, did not show a statistically significant impact on the ability to extubate early (Table 8).

**Table 8:** Factors associated with extubation within 24 hours (n = 56).

Variable	Category	Early extubation, n (%) (n = 38)	Delayed, n (%) (n = 18)	Test	p-value
Age (months)	Mean ± standard deviation	2.41 ± 3.44	6.33 ± 6.71	Mann-Whitney	0.002
Sex	Male	21 (55.3)	12 (66.7)	Chi-square	0.418
	Female	17 (44.7)	6 (33.3)		
Presenting symptom	Respiratory distress	21 (55.3)	6 (33.3)	Chi-square	0.051
	Feeding difficulty	11 (28.9)	3 (16.7)		
	Failure to thrive	3 (7.9)	6 (33.3)		
	Cyanosis	3 (7.9)	3 (16.7)		
Laryngotracheobronchoscopy findings	Aryepiglottoplasty	9 (23.7)	6 (33.3)	Chi-square	0.067
	Epiglottopexy	20 (52.6)	3 (16.7)		
	Resection of the arytenoid mucosa	5 (13.2)	6 (33.3)		
	Combined	4 (10.5)	3 (16.7)		

Multivariate logistic regression models were constructed for each outcome, including variables that were significant on univariate analysis (age, failure to thrive, and type 3 laryngomalacia). On multivariate logistic regression analysis, increasing age (adjusted odds ratio = 1.32, p = 0.006) and failure to thrive (adjusted odds ratio = 4.86, p = 0.034) were associated with a higher likelihood of requiring a second intervention. Type 3 laryngomalacia was associated with increased odds of tracheostomy (adjusted odds ratio = 9.80, p = 0.024). No independent predictors were identified for relief from stridor or delayed extubation (Table 9).

**Table 9:** Multivariate logistic regression analysis of clinical outcomes (n = 56). Highly significant values are in bold.

Outcome	Predictor	Adjusted odds ratio	95% confidence interval	p-value
Relief from stridor	Age (months)	0.88	0.74-1.05	0.162
	Failure to thrive	0.43	0.05-3.54	0.435
	Type 3 laryngomalacia	0.45	0.08-2.61	0.371
Delayed extubation	Age (months)	1.18	0.98-1.41	0.078
	Failure to thrive	1.92	0.38-9.64	0.431
	Type 3 laryngomalacia	1.36	0.30-6.10	0.689
Second intervention	Age (months)	1.32	1.08-1.62	0.006
	Failure to thrive	4.86	1.12-21.1	0.034
	Type 3 laryngomalacia	2.74	0.63-11.9	0.178
Tracheostomy	Age (months)	1.41	0.89-2.25	0.141
	Failure to thrive	6.20	0.86-44.5	0.071
	Type 3 laryngomalacia	9.80	1.35-71.2	0.024

## Discussion

Laryngomalacia is widely recognized as the most common cause of congenital infantile stridor, accounting for approximately 60-75% of cases [1]. Although the majority of affected infants follow a benign course with spontaneous resolution by two years of age, a subset (10-15%) develops severe disease requiring surgical intervention due to complications such as upper airway obstruction, failure to thrive, and cardiopulmonary compromise [1]. The present study focuses on this severe subset and evaluates demographic profile, clinical characteristics, surgical outcomes, and factors influencing postoperative recovery and need for tracheostomy.

In the current study, the mean age at presentation was 3.67 ± 5.03 months, with a median age of two months. Most patients (91.1%) presented within the first 10 months of life, which is consistent with the known natural history of laryngomalacia, where symptoms typically manifest early in infancy. A male predominance was observed (58.9%), aligning with previous reports that have demonstrated a higher incidence of laryngomalacia among male infants. Respiratory distress was the most common presenting symptom (48.2%), followed by feeding difficulty (25%) and failure to thrive (16.1%), reflecting the spectrum of clinical severity described in severe laryngomalacia (Table 1).

Laryngotracheobronchoscopic evaluation revealed that type 2 laryngomalacia was the most frequent anatomical subtype (41.1%), followed by type 1 (26.8%) and type 3 (19.6%). Combined types were identified in 12.5% of patients. These findings are consistent with previous studies reporting a predominance of type 2 collapse in surgically treated cohorts [8]. Accurate endoscopic assessment remains critical, not only for classification but also for tailoring the surgical approach. Similar to earlier reports, the use of rigid endoscopy under light general anesthesia with spontaneous respiration allowed optimal visualization of supraglottic collapse and facilitated assessment of distal airway pathology.

Supraglottoplasty remains the cornerstone of surgical management for severe laryngomalacia. In the present study, aryepiglottoplasty was the most frequently performed procedure (41.8%), either alone or in combination with other techniques. Resection of the arytenoid mucosa and epiglottopexy were employed selectively based on anatomical findings. Importantly, the choice of surgical technique did not significantly influence relief from stridor, delayed extubation, need for tracheostomy, or requirement for second intervention. This supports prior observations by van der Heijden et al. [9] and Hoff et al. [10], who reported that outcomes were more closely related to disease severity and comorbid factors than to the specific surgical method used.

Postoperative outcomes in this study were favorable, with 80.4% of patients achieving relief from stridor, comparable to success rates reported by Denoyelle et al. (79%) [11] and Drummond et al. [12]. Early extubation within 24 hours was achieved in 67.9% of patients, while delayed extubation occurred in 17.9%. Notably, patient age emerged as a consistent and significant predictor across multiple outcomes. Younger patients were more likely to experience relief from stridor, achieve early extubation, avoid second intervention, and have uncomplicated postoperative courses. These findings suggest that earlier surgical intervention may confer improved functional outcomes, possibly due to greater airway compliance and fewer secondary physiological consequences of prolonged obstruction.

Failure to thrive was strongly associated with poorer outcomes. It was significantly more common among patients who failed to achieve stridor relief (45.5%), required a second intervention (50%), or underwent tracheostomy (66.7%). This observation reinforces the concept that failure to thrive is not merely a symptom but a marker of advanced disease severity, as previously suggested by van der Heijden et al. [9]. These patients likely represent a subgroup with prolonged hypoxia, increased work of breathing, and impaired feeding efficiency.

The need for tracheostomy was low in the present study (5.4%), which compares favorably with previously published series. Importantly, all patients requiring tracheostomy had type 3 laryngomalacia, demonstrating a highly significant association between this anatomical subtype and airway compromise. This finding underscores the aggressive nature of posterior supraglottic collapse and mirrors reports in the literature identifying type 3 laryngomalacia as a predictor of surgical failure and prolonged airway dependence [12-14].

Second interventions were required in 14.3% of patients, a rate similar to other studies evaluating severe disease [15]. Older age at presentation and failure to thrive were significant predictors, while anatomical subtype and surgical technique were not. Unlike van der Heijden et al. [9], who managed failures primarily with revision supraglottoplasty, the present study employed tracheostomy in select cases, reflecting individualized decision-making based on disease severity and associated conditions.

A notable finding of this study is the absence of intraoperative or postoperative complications. This contrasts with the report by Mandour et al. [16], who observed complications in 7.4% of cases, including supraglottic stenosis and transient aspiration. Furthermore, while some authors advocate unilateral aryepiglottoplasty to reduce complication rates, the current study demonstrates that bilateral procedures using simple cautery can be performed safely, without increased morbidity. This also differs from studies favoring laser-based techniques for epiglottopexy, suggesting that cost-effective alternatives may yield comparable outcomes when performed judiciously [11].

Limitations

Despite encouraging results, this study is limited by its single-center design and lack of long-term follow-up beyond early postoperative outcomes. Additionally, comorbidities and synchronous airway lesions were not analyzed in depth, which may further influence outcomes.

## Conclusions

The findings of this study support surgical intervention as a safe and effective treatment for severe laryngomalacia, with high success rates and minimal morbidity. Younger age at intervention and absence of failure to thrive are key predictors of favorable outcomes, while type 3 laryngomalacia remains a strong predictor of tracheostomy. This study emphasizes the importance of individualized anatomical assessment and early intervention.
